# The role of prestimulus activity in visual extinction^☆^

**DOI:** 10.1016/j.neuropsychologia.2013.05.005

**Published:** 2013-07

**Authors:** Maren Urner, Margarita Sarri, Jessica Grahn, Tom Manly, Geraint Rees, Karl Friston

**Affiliations:** a UCL Institute of Cognitive Neuroscience, London WC1N 3AR, UK; b Wellcome Trust Centre for Neuroimaging, UCL Institute of Neurology, London WC1N3BG, UK; c UCL Institute of Child Health, London WC1N 1 EH, UK; d Department of Psychology, Brain and Mind Institute, University of Western Ontario, London, ON, Canada N6A 5B7; e MRC Cognition and Brain Sciences Unit, Cambridge CB2 7 EF, UK

**Keywords:** Visual extinction, Spatial neglect, Prestimulus activity, Predictive coding, Dynamic causal modelling (DCM), Visual processing

## Abstract

Patients with visual extinction following right-hemisphere damage sometimes see and sometimes miss stimuli in the left visual field, particularly when stimuli are presented simultaneously to both visual fields. Awareness of left visual field stimuli is associated with increased activity in bilateral parietal and frontal cortex. However, it is unknown why patients see or miss these stimuli. Previous neuroimaging studies in healthy adults show that prestimulus activity biases perceptual decisions, and biases in visual perception can be attributed to fluctuations in prestimulus activity in task relevant brain regions. Here, we used functional MRI to investigate whether prestimulus activity affected perception in the context of visual extinction following stroke. We measured prestimulus activity in stimulus-responsive cortical areas during an extinction paradigm in a patient with unilateral right parietal damage and visual extinction. This allowed us to compare prestimulus activity on physically identical bilateral trials that either did or did not lead to visual extinction. We found significantly increased activity prior to stimulus presentation in two areas that were also activated by visual stimulation: the left calcarine sulcus and right occipital inferior cortex. Using dynamic causal modelling (DCM) we found that both these differences in prestimulus activity and stimulus evoked responses could be explained by enhanced effective connectivity within and between visual areas, prior to stimulus presentation. Thus, we provide evidence for the idea that differences in ongoing neural activity in visually responsive areas prior to stimulus onset affect awareness in visual extinction, and that these differences are mediated by fluctuations in extrinsic and intrinsic connectivity.

## Highlights

• Prestimulus activity in visual extinction affects perception.
• The BOLD signal in two visual areas is indicative of perception in bilateral trials.
• Intrinsic and extrinsic connectivity is elevated prior to perceived trials.

## 1 Introduction

### 1.1 The phenomenon of visual extinction

Visual extinction is commonly observed after right parietal damage. Patients with visual extinction perceive unilateral stimuli presented either in the left *or* the right visual field, but sometimes miss a stimulus in the left visual field during bilateral simultaneous presentation. Awareness of these left visual field stimuli is effectively “extinguished” by the stimulus in the right visual field. Visual extinction therefore offers a rare opportunity to study the neural correlates of perceptual awareness and unconscious processing.

### 1.2 How does visual extinction relate to spatial neglect?

The nosology of visual extinction is not clear. It could either represent a component, or mild form, of the classical visuospatial neglect syndrome (Heilman, Watson, & Valenstein, 1994; Rafal, 1994; Vallar, 1993) or a completely different type of visuospatial attention deficit (Umarova et al., 2011). Some data suggest a dissociation between the two syndromes (Hillis et al., 2006; Vallar, Rusconi, Bignamini, Geminiani, & Perani, 1994; Vossel et al., 2011), whereas others emphasise the similarity, especially when the lesions are clustered in the inferior parietal lobule (Posner, Walker, Friedrich, & Rafal, 1984; Rees et al., 2000, Vallar et al., 1994; Vuilleumier & Rafal, 2000). Umarova et al. (2011) compared the activation patterns of acute stroke patients with neglect and visual extinction during visuospatial processing and found an increased activation in the left prefrontal cortex only for patients with extinction. These results suggest that visual extinction and neglect are separate syndromes. However, this study used only unilateral stimuli and did not identify the areas involved in the extinction of the left stimulus during bilateral stimulation. Interestingly, the right inferior parietal cortex has been implicated in the simultaneous processing of bilateral targets (in animal studies (Lynch & Mclaren, 1989) and healthy participants (Ciçek, Gitelman, Hurley, Nobre, & Mesulam, 2007).

### 1.3 Mechanisms of visual extinction

Several previous studies have investigated the neural mechanisms of visual extinction, using bilateral and unilateral stimuli. Essentially, two different approaches have been employed. The first approach investigates residual cortical processing of the extinguished stimulus by comparing responses in bilateral extinguished trials with responses in unilateral right trials; i.e., trials with different physical properties that lead to the same behavioural response. Contrasting these experimental conditions using functional MRI shows that the extinguished stimulus in the left visual field activates early visual cortex, as well as the extrastriate visual cortex in the damaged right hemisphere, e.g. (Driver, Vuilleumier, Eimer, & Rees, 2001; Rees et al., 2000, 2002b; Rees, Kreiman, & Koch, 2002a; Vuilleumier et al., 2002; Vuilleumier et al., 2010). A cross modal study using the same paradigm with tactile information reported activation of primary sensory cortex (S1) in response to extinguished stimuli (Sarri, Kalra, Greenwood, & Driver, 2006). These results provide a potential explanation for the unconscious processing assessed using indirect measures such as priming, e.g. (Baylis, Driver, & Rafal, 1984; Berti, Rizzolatti, & Umana, 1987; Driver et al., 2001; Ladavas, Paladini, & Cubellli, 1993; Vuilleumier et al., 2002, 2010).

The second approach examines the neural correlates of awareness by comparing seen and unseen stimuli during bilateral presentation; i.e., trials with the same physical properties leading to different behavioural responses. Converging evidence from several studies supports the idea that the interplay between posterior visual areas and fronto-parietal circuits is crucial for a visual stimulus to reach awareness, e.g. (Driver et al., 2001; Rees et al., 2002a, 2002b). Thus, it has been suggested that a pathological bias in attention towards the ipsilesional visual field leads to the “extinction” of the contralesional stimulus from awareness during bilateral stimulation. This is in line with the observation that the colour and form of the extinguished stimulus can still be processed to a certain extent. In short, the parietal damage might compromise spatial awareness and responding, rather than disrupting early visual processing.

### 1.4 Prestimulus activity affects perception

It is well known that ongoing or intrinsic neuronal activity influences subsequent evoked responses. Furthermore, prestimulus activity has been related to systematic variations in behaviour and thus is functionally significant. For example, Fox, Snyder, Vincent, and Raichle (2007) found that 74% of spontaneous trial-to-trial variability in button press force can be accounted for by ongoing fluctuations in the intrinsic activity in somatosensory cortex. Similarly, correlations between ongoing fluctuations of brain activity and perception are observed across different paradigms and different species (Giesbrecht, Jongen, Smulders, & Merckelbach, 2006; Hesselmann, Kell, Eger, and Kleinschmidt, 2008a; Hesselmann, Kell, and Kleinschmidt, 2008b; Ress, Backus, & Heeger, 2000). Fluctuations in prestimulus activity in visual areas measured with EEG and MEG influences the detection of upcoming stimuli (Mathewson, Gratton, Fabiani, & Beck, 2009; Wyart & Tallon-Baudry, 2009). Specifically, alpha activity in somatosensory areas might play a crucial role in optimising neuronal processing, thereby influencing behaviour (Haegens, Händel, & Jensen, 2011). In addition, functional MRI results suggest that the BOLD signal in a cortical area preferentially responding to faces is higher preceding experimental trials that are perceived as faces compared to vases using an ambiguous figure (Hesselmann et al., 2008a). In motion coherence tasks, BOLD signals in motion-responsive brain areas are higher before trials that are perceived as showing coherent compared to random motion (Hesselmann et al., 2008b). Finally, a recent functional MRI study extended the investigation of fluctuations in ongoing brain activity to the domain of cognitive control: prestimulus activity in several task relevant regions – including higher cognitive areas – scales with the size of the Stroop effect (Coste, Sadaghiani, Friston, & Kleinschmidt, 2011). In sum, there is strong evidence that endogenous variations in prestimulus neuronal activity bias subsequent perceptual decisions.

### 1.5 Can we analyse visual extinction using prestimulus activity?

Here, we set out to answer the question *how* it is possible that patients with visual extinction sometimes see and sometimes miss the left stimulus during bilateral stimulation. Our strategy was to compare prestimulus BOLD signals before bilateral visual stimulus presentation depending on whether the trial was subsequently categorised as a “bilateral seen” or as a “bilateral unseen” trial; in other words, whether the patient failed to detect the stimulus in the left visual field. We focused on visually response areas and used a simple detection paradigm with bilateral and unilateral face stimuli. First, we identified visually responsive areas in a patient showing visual extinction. Second, we compared prestimulus activity in these regions during bilateral stimulation with and without extinction. Finally, we used dynamic causal modelling (DCM) (Friston, Harrison, & Penny, 2003) to examine whether changes in the coupling or excitability of these regions could explain both prestimulus activity and subsequent differences in stimulus bound responses. Specifically, we investigated whether extinction might be mediated by a difference in intrinsic (within area), or extrinsic (between areas), effective connectivity, i.e. the causal influences that neural units exert over one another (Friston, 1994), or sensitivity to neuronal afferents. DCM is the method of choice for our question because it tests hypotheses or models that are cast in terms of directed connections among neuronal populations. This contrasts with less informed approaches – such as functional connectivity – that simply measure (undirected) correlations between haemodynamic responses at different points in the brain.

## 2 Material and methods

### 2.1 Participant

One male patient (IPJ) aged 66 with left visual extinction (following a right parietal stroke, see Fig. 2) gave informed consent to participate in the study. The participant showed left visuospatial neglect on four standard clinical measures – see Section 2.2.1. Functional imaging was conducted 3 years and 4 months post-stroke. IPJ was suited for in-depth study as he had a structurally intact visual cortex in the right hemisphere, despite suffering from enduring visual extinction on clinical confrontation and formal computerised testing. However, he showed lower left quadrant visual field impairment. Therefore, all experimental stimuli were presented in the upper visual quadrants.

### 2.2 Design and procedure

The experiment was approved by the local ethics committee.

#### 2.2.1 Neuropsychological testing

Prior to functional imaging, IPJ was tested for clinical signs of visual extinction by confrontation. In addition, he was presented with bilateral, unilateral left and unilateral right visual stimuli outside of the scanner to titrate the different parameters for the scanning sessions. The criteria defined by Vallar et al. (1994) (i.e. >30% misses of left events during bilateral stimulation, but<20% misses of single left events during unilateral stimulation) were used. In addition, IPJ performed three standard clinical neuropsychological measures to test for signs of visuospatial neglect: in the cancellation task, IPJ was presented with an A4 sheet of paper containing circles and crosses. Half of these contained a small gap, which had to be crossed out (15 on each side, i.e. 30 in total). Typically, patients with neglect fail to cancel targets located on the left side of the page.

During the line bisection task, e.g. (Wilson, Cockburn, & Haligan, 1987), IPJ was presented with three 18 cm lines printed in the middle of A4 sheets of paper and was asked to put a mark where he thought the middle of each line was. Neglect patients tend to underestimate the leftmost side of the line, thus making errors by deviating rightwards from the true midpoint. In the lateral preference task, which measures spontaneous lateral attentional biases, the patient was shown 10 pairs of virtually arranged, identical, left-right mirror-reversed chimeric face stimuli – joining together left and right halves of the same face posing different neutral or happy expressions. The patient was asked to judge whether the upper or bottom face looked happier. Right hemisphere damaged patients with left neglect typically select the face that is smiling on the right side of the display, e.g. (Sarri, Ruff, Rees, & Driver, 2010), which is the opposite for healthy participants, e.g. (Mattingley, Pierson, Bradshaw, Phillips, & Bradshaw, 1993).

#### 2.2.2 fMRI paradigms

After the behavioural data had been analysed, IPJ was tested during two scanning paradigms using functional MRI (on separate days), which we refer to as the “extinction paradigm” and the “stimulus localiser”. During both paradigms he was asked to fixate centrally and to respond with the right hand on a keypad.

##### 2.2.2.1 Extinction paradigm (event related design)

**Fig. 1:**
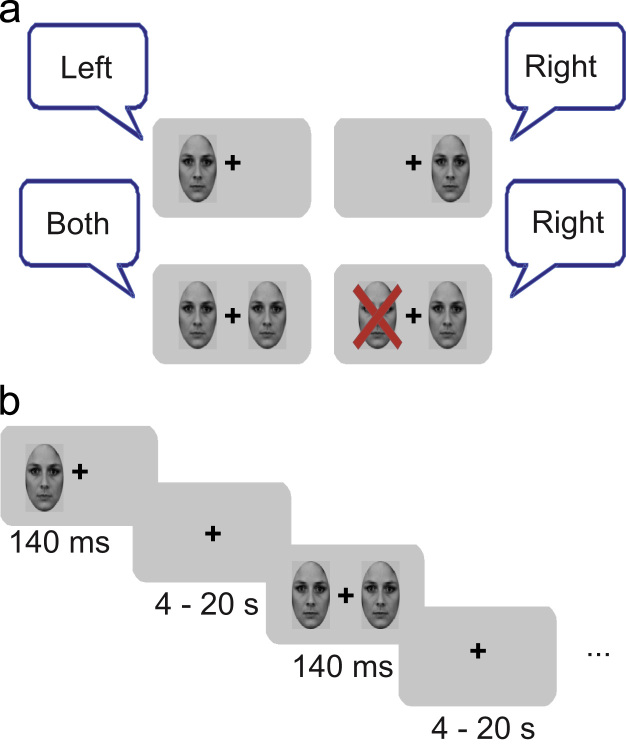
The extinction paradigm. (a) Facial stimuli were presented unilaterally in either the left or right visual fields (upper row) or bilaterally (lower row). Depending on the response of the patient, trials were categorised after scanning as bilateral seen (BS) (lower row, left) and bilateral unseen (BU) (lower row, right) trials. (b) Stimuli were presented for 140 ms (or 120 ms respectively during the later blocks due to learning effects of the patient) and were segregated by an intertrial interval ranging between 4 and 20 s.

**Fig. 2:**
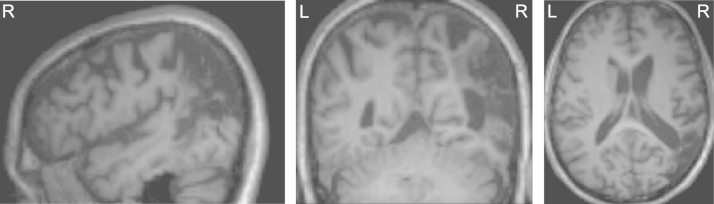
Right parietal lesion. T1-weighted structural MRI scan acquired during the first of two scanning sessions where the pre-existing right parietal lesion is clearly apparent.

Each trial of the extinction paradigm comprised the presentation of faces on the left, the right or both sides. Stimuli were presented for 140 ms (run 1–6) or 120 ms (run 7–9). The duration was shortened during the last three runs to ensure an equal number of bilateral seen (BS) and bilateral unseen trials (BU), as IPJ improved in terms of visual detection. His task was to indicate where he saw a stimulus or stimuli respectively. The conditions were presented in random order and the inter-trial interval was randomised to minimise anticipation, ranging between 4 and 20 s. See Fig. 1 for a visual description of the extinction paradigm. Each run comprised 35 trials, with 23 bilateral stimulus presentations and 12 unilateral presentations (i.e. six, for each side). IPJ completed nine runs divided over two scanning sessions (five in the first session), resulting in 207 bilateral and 54 trials for each side respectively.

##### 2.2.2.2 Stimulus localiser (block design)

Each trial of the stimulus localiser entailed the presentation of faces, objects or scrambled images on the left or right side. To elicit detectable responses in visual areas, stimuli were presented for 250 ms with an inter-stimulus interval of 500 ms. Based on previous experiments on visual extinction, we used longer stimulus presentation times (compared to the extinction paradigm) thereby increasing the efficiency or sensitivity of detecting visually responsive areas in the lesioned brain. IPJ completed two runs each consisting of 12 blocks during which each stimulus (faces left or right, objects left or right, scrambled images left or right) appeared twice (i.e. 12 trials per block). Each block was followed by a 6 s break (i.e. blank screen). To ensure fixation throughout, the task was to press a button whenever the fixation cross turned red. Note that, unlike the event related extinction paradigm, this paradigm was a more efficient and longer block design that only presented unilateral visual stimuli. This enabled us to identify visually responsive areas for subsequent analysis in an efficient way. Furthermore, because we were particularly interested in the mediation of extinction in early visual cortex (providing ascending sensory information to higher category selective regions), we averaged over all stimuli types in the localiser to define functionally preserved visual responses at lower levels in the visual hierarchy.

##### 2.2.2.3 Stimuli

All stimuli were presented at the same location in the upper quadrants of the visual field, subtending 4.91×6.70 of visual angle. Face stimuli were taken from a face database provided by the Karolinska Institute, Stockholm, Sweden (Oosterhof & Todorov, 2008), and were cropped, resized, and presented in greyscale. Scrambled images were derived from the object and face images via a random exchange of picture elements organised in a 20×20 matrix.

### 2.3 fMRI data acquisition

A 3T Trio MRI scanner (Siemens Medical Systems, Erlangen, Germany) with a standard head coil was used to acquire functional data with a standard echo planar imaging (EPI) sequence (matrix size 64×64; field of view 192×192 mm; in plane resolution 3×3 mm; 32 slices in descending acquisition order; slice thickness 3 mm; echo time 30 ms; TR 2 s). IPJ attended two scanning sessions separated by 1 week. During both sessions, fieldmaps were acquired to correct for geometric distortions in the EPI images due to inhomogeneities of the magnetic field. Finally, a structural T1-weighted scan was acquired during each session (field of view 256×240 mm; in-plane resolution 1×1 mm; 176 sagittal slices of thickness 1 mm; echo time 2.98 ms). Each run included five dummy volumes that were discarded during the data analysis to allow for T1 equilibration.

### 2.4 Data analysis

#### 2.4.1 Behavioural data

Data from the extinction paradigm were analysed with regard to correct trials and reaction times. These were compared among different conditions using repeated measures ANOVAs.

#### 2.4.2 fMRI data

Functional data were analysed using SMP12 (http://www.fil.ion.ucl.ac.uk/spm/software/spm12/). Pre-processing of the data involved realignment of each scan to the first scan of each run, coregistration of the functional data to the structural data of each day and, finally, coregistration of the structural scan of the second day, to co-register all the functional images. The functional data were smoothed with an 8 mm Gaussian kernel after spatial normalisation to the MNI template brain. The data were filtered with a standard 128-s cut-off, high-pass filter to remove low-frequency drifts (including differences between runs), while preserving as much variance due to spontaneous fMRI fluctuations as possible (Cordes et al., 2001). Statistical tests were family wise error rate corrected (FWE) for multiple comparisons at *p*<0.05 or uncorrected at *p*<0.001 across the entire brain.

##### 2.4.2.1 Extinction paradigm

The time-series of each functional run were analysed using a standard general linear model (GLM) including eight regressors for the four conditions or trial types of interest: right and left unilateral trials and bilateral trials on which the stimulus was seen (BS) or unseen (BU): each condition had two regressors, one for the prestimulus baseline and one for the stimulus evoked responses. The prestimulus baseline was modelled as a 6 s long period starting 7 s before stimulus onset (allowing a 1 s gap between baseline and stimulus presentation). The choice of 7 s was based upon informal model comparisons, using models of sustained prestimulus activity starting 3 s and 5 s before stimulus onset (not reported) and heuristics based upon the timescale of fluctuations in resting state fMRI studies. These fluctuations have a characteristic length of about 10 s, which places an upper bound on the duration of sustained endogenous activity.

The evoked responses were modelled as standard event-related stick functions. Note that the prestimulus baseline and event related response regressors for each trial type were necessary correlated, because one precedes the other. However, because the haemodynamic response function peaks between 4 and 6 s, the activity modelled by the two regressors could be estimated with reasonable efficiency. We did not orthogonalise these regressors, which means that any significant prestimulus baseline effects discovered cannot be explained by event related differences.

Stimulus functions were convolved with a canonical haemodynamic response function to provide regressors for a standard general linear model (GLM). Movement parameters in the three directions of motion and three degrees of rotation were included as confounding regressors of no interest. Contrasts of parameters of the effect of interests were estimated over all nine task runs. The associated statistical parametric maps (SPMs) were used to test for differences in the neural activity during the prestimulus period of BS and BU trials.

##### 2.4.2.2 Stimulus localiser

The time-series of both functional runs were analysed with a standard GLM comprising six regressors modelling the effects of faces, objects and scrambled images for left and right side, using even-related regressors. Again, movement parameters were included as confounding regressors of no interest. Contrasts of parameters were estimated over both two task runs. The resulting SPMs were used to test for differences in the neural responses between right and left visual field stimulation to identify regions showing visual responses to lateralised stimuli.

##### 2.4.2.3 Peri-stimulus time histograms (PSTH)

In order to quantify the time course of the BOLD activity in the regions of interest (ROI) showing differences between seen and unseen trials (i.e. BS vs. BU), we estimated event related responses in these ROIs using a finite impulse response (FIR) convolution model. The parameters of the corresponding GLM report BOLD activity in successive time bins of 2 s of peristimulus time (in our case). We evaluated event related responses over all nine runs from 7 s before to 9 s after stimulus presentation.

##### 2.4.2.4 Dynamic causal modelling (DCM)

The standard SPM analyses above localised (visually responsive) brain areas that show higher activity before BS compared to BU trials. Our hypothesis was that perception depends upon prestimulus baseline activity and that this activity depends upon fluctuations in extrinsic or intrinsic connectivity. In the final analyses, we used dynamic causal modelling to determine whether differences in connectivity between seen and unseen trials were intrinsic to the visual regions showing prestimulus baseline effects and/or in the extrinsic connections between these regions.

Our comparisons of effective connectivity were based on Bayesian model comparison using (deterministic) dynamic causal modelling (DCM) (Penny, Stephan, Mechelli, & Friston, 2004). To test for differences in effective connectivity we concatenated the data of all nine runs and used three regressors: one for the prestimulus baseline of all bilateral trials (using 7 s boxcar functions: the duration of the prestimulus period was extended to 7 s, to ensure that prestimulus conditions were maintained until the stimulus arrived), one for the stimulus onset of all bilateral trials (using a standard event related stimulus function) and one to indicate whether the stimulus was seen or not (i.e. using the same boxcar function as for the first regressor but only for BS trials).

We created 16 models corresponding to a 4×4 factorial design with two factors. All models included reciprocal extrinsic connections between the two visual areas of interest (the areas are referred to as “right” and “left” subsequently), which were driven by the prestimulus and stimulus onset effects described in Sections 2.4.2.1 and 2.4.2.2. The first factor was extinction-dependent differences in intrinsic connections of the two regions (with the four levels: both, left, right, or neither), while the second factor was differences in extrinsic connections between those two regions (with the four levels: both, left-to-right, right-to-left, or neither). Crucially, both the prestimulus and stimulus related driving effects were identical for seen and unseen trials. The only difference between seen and unseen trials was mediated by a prestimulus effect that modulated connections within (intrinsic) or between (extrinsic) the two regions. In other words, the extinction of the left stimulus could only be explained by a difference in (intrinsic or extrinsic) connectivity or sensitivity to presynaptic inputs that was established before the arrival of the stimulus.

All 16 models were fitted to the concatenated time series of the extinction runs using standard variational Bayesian model inversion. The relative evidence for each model was approximated with variational free energy to provide the posterior probability of each model (assuming uniform priors over subsets of families of models that were compared) (Friston, Mattout, Trujillo-Barreto, Ashburner, & Penny, 2007, Stephan, Penny, Daunizeau, Moran, & Friston, 2009). We used a two-step heuristic search for the best model: First, we assessed the contribution of changes in intrinsic connectivity by assessing the posterior probability for the four different families of intrinsic connection strength changes (effectively averaging over our uncertainty about putative changes in extrinsic connections). We then compared the four different extrinsic models within the winning intrinsic family.

Finally, we examined the modulation of connections, i.e. changes in connection strength, using the parameter estimates for the intrinsic and extrinsic connections of the winning model. Note that in this DCM, the modulatory or bilinear effects are modelled by additive changes to the connection strengths. This means that the modulatory values alongside the connections in Fig. 7 should be added to the coupling parameters associated with each connection. The ensuing modulation of connections by a prestimulus effect presupposes an endogenous fluctuation in the local synaptic processes that determine effective connectivity. In other words, the prestimulus effect is an effect on coupling strength (quantified by DCM) that causes changes in neuronal activity (quantified by SPM).

## 3 Results

### 3.1 Patient showed signs of visual extinction

Four typical clinical neuropsychological measures of neglect were used to test for signs of visual extinction. In the cancellation task IPJ missed three targets on the left side and none on the right side. In the line bisection task, IPJ's mean deviation error toward the right when indicating the middle of the line was 3.3 cm. In the lateral preference task the patient chose faces with the smile on the right side in nine out of 10 cases. During confrontation IPJ missed the stimulus presented in his left visual field in nine out of 10 bilateral trials. He did not miss any of the unilateral left trials. Thus, he fulfilled the criteria defined by Vallar et al. (1994).

### 3.2 Stimulus localiser activated visual areas

**Fig. 3:**
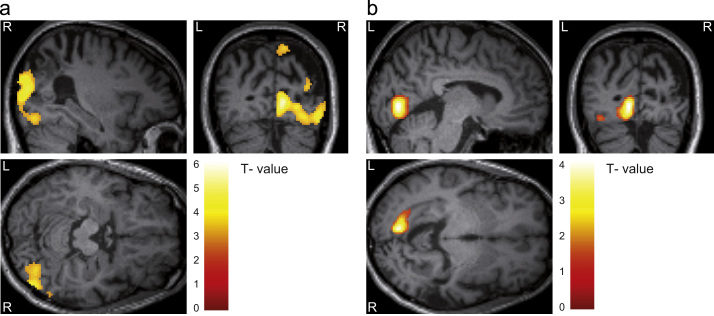
Stimulus localiser activated visual areas in both hemispheres. (a) Activations due to stimulation of the left visual field were confined to the right hemisphere. Images are displayed at *p*<0.001, uncorrected for illustration purpose. (b) Activations due to stimulation of the right visual field were confined to the left hemisphere and showed much less distributed pattern. Images are displayed at *p*<0.01, uncorrected for illustration purpose.

Comparing BOLD signals for stimulus presentation in the left visual field (i.e. independent of stimulus type) to those for presentation in the right visual field, we found activations in three regions in the right hemisphere (see Table 1), including primary visual areas and precuneus. The opposite contrast, testing for regions that were more active during presentation of a stimulus in the right visual field, revealed activation of left primary visual cortex. However, the activation was much more confined. See Fig. 3 and Table 1 for detailed results.

### 3.3 Extinction paradigm produced unseen trials

**Fig. 4:**
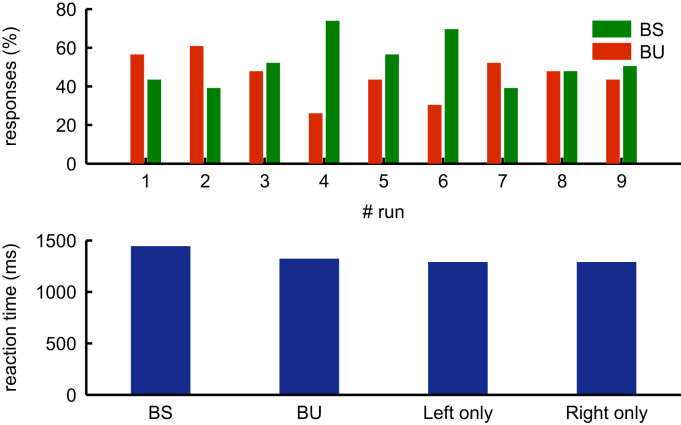
Behavioural results of the extinction paradigm. (A) Percentages of BS and BU trials for all nine runs. (B) Reaction times for the four trial types (see text) averaged over all nine runs.

Averaged over the nine runs of the extinction paradigm, IPJ missed 45% of bilateral trials (corresponding to 94 out of 207 trials) – these are the BU trials. There was no significant difference between BS and BU trials over the nine runs (F(1, 8) =.97, *p*=.35). He reported seeing 94% (50 out of 54 trials) of unilateral left trials, and 98% (53 out of 54 trials) of unilateral right trials. The difference in seen unilateral trials was not significant over the nine runs (F(1, 8)=4.00, *p*=0.08). Average response times for the BS trials were longest, with unilateral trials being faster than bilateral trials; however, reaction times did not differ significantly between the different trials (F(3, 24)=1.70, *p*=.20). See Fig. 4 for the details of the responses and reaction times.

### 3.4 Prestimulus activity in visually responsive areas affects perception

**Fig. 5:**
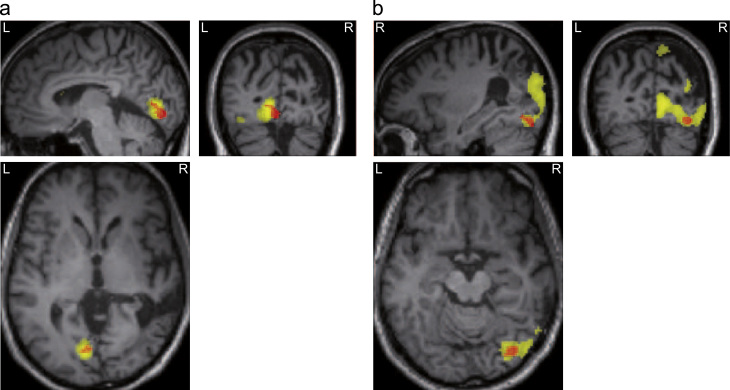
Visually responsive areas are more active before bilateral seen trials. Overlay between lateralised visually evoked responses during the stimulus localiser (yellow) and the prestimulus baseline effects revealed by the extinction paradigm (red), showing the areas that are more active before BS compared to BU trials. (A) Left hemisphere. (B) Right hemisphere. All overlays are displayed at *p*<0.005, uncorrected for illustration purpose. (For interpretation of the references to color in this figure caption, the reader is referred to the web version of this article.)

We identified regions showing higher activity before BS compared to BU trials by comparing the BOLD signal between these two conditions in a 6 s prestimulus baseline window starting 7 s before stimulus presentation. Crucially, we found an overlap with visual areas that were activated by the stimulus localiser in both hemispheres: BA 19/ occipital inferior right cortex (MNI *x*=36, *y*=−78, *z*=−16, *t*=3.32, *p*<0.001 uncorrected) and BA 17/ calcarine sulcus left (MNI *x*=−4, *y*=−86, *z*=−8, *t*=3.28, *p*<0.001 uncorrected). The overlap between the visual responses to bilateral stimuli and the localiser stimuli was substantial: 82% (65 out of 79 voxels) of the BS–BU activation in the right hemisphere overlapped with the activation due to the stimulus localiser in the right hemisphere, 47% (69 out of 147 voxels) of the BS–BU activation in the left hemisphere overlapped with the activation due to the stimulus localiser in the left hemisphere. See Fig. 5.

In addition, an exploratory analysis (using an uncorrected threshold of *p*<0.001) revealed several regions showing an effect in the same direction, including activity differences in the brain stem and parietal cortex. See Table 2 for an overview. The opposite contrast, i.e. higher activity before BU vs. BS trials, revealed no region that would survive FWE correction. The closest was a right inferior frontal area (MNI *x*=54, *y*=16, *z*=4, *t*=3.43, *p*<0.001, uncorrected).

### 3.5 Time-course of responses to seen and unseen trials

**Fig. 6:**
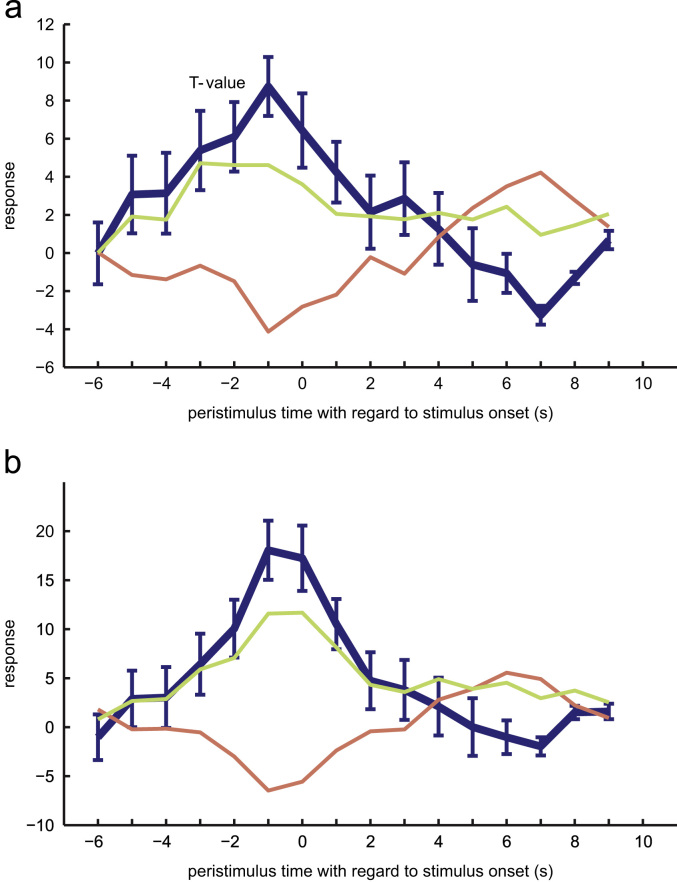
Peristimulus time courses show difference before stimulus onset. The time courses of the two visually responsive areas showed a differential activity during baseline before stimulus presentation depending on whether the subsequent bilateral stimulus is seen. Plotted are the difference with SD (blue) and the time course for BS (green) and BU (red) individually. (A) ROI in right occipital inferior cortex. (B) ROI in left calcarine sulcus. (For interpretation of the references to color in this figure caption, the reader is referred to the web version of this article.)

To quantify the prestimulus fluctuations in BOLD responses, we used an FIR model for responses in the two visual areas that showed increased activity before BS compared to BU trials. Both ROIs show a distinct increase in their haemodynamic response before stimulus presentation, which starts to diverge between seen and unseen trials as early as 5 s before stimulus onset. See Fig. 6 for details.

### 3.6 Perception depends on the coupling between visual areas

Having identified two visually responsive areas that showed increased activity preceding BS trials, compared to BU trials, we next asked whether the connectivity within and between those two regions differed before stimulus exposure. The models tested differed in terms of where differences in connectivity were expressed depending on whether a bilateral trial was seen or not. Sixteen models as described in Section 2.4.2.4 were fitted and compared in terms of the posterior probabilities. The first comparison between intrinsic families showed that we could be 99% confident that there was an effect on intrinsic connections and 73% confident that both visual areas were involved (although there was a 26% probability that only the left area was affected). Following this comparison, we compared the four models within the winning intrinsic family (were both intrinsic connections changed). This comparison showed that we could be 99% sure that there was a change in extrinsic connections and 68% confident that both efferent and afferent connections to the lesioned hemisphere were involved (although there was a 30% chance that just the right to left extrinsic projection changed).

**Fig. 7:**
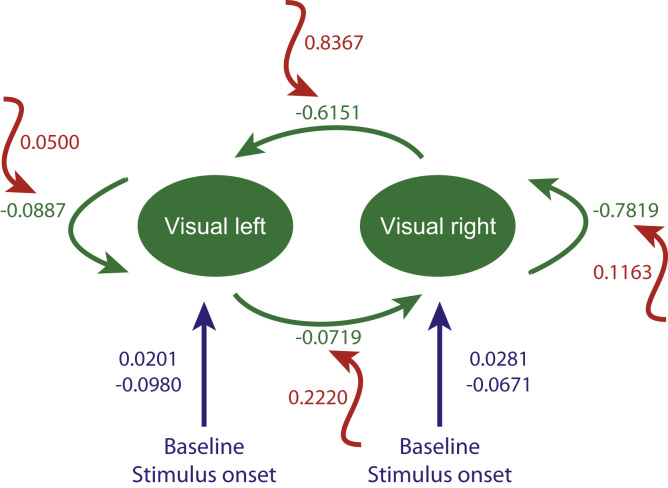
Differences in effective connectivity before bilateral seen trials. The winning model including its inputs (blue), intrinsic and extrinsic connectivity (green) and modulations (red) is shown. The numbers describe the parameter weights. Effective connectivity was increased for all four intrinsic and extrinsic connections prior to bilateral seen trials. Visual left=calcarine sulcus, visual right=inferior occipital cortex. (For interpretation of the references to color in this figure caption, the reader is referred to the web version of this article.)

Having selected the most plausible model, we looked at the differences in effective connection strength between seen and unseen trials. For BS trials, effective connectivity within and between the two areas increased. See Fig. 7 for details. Crucially, all intrinsic and extrinsic effective connection strengths were elevated prior to seen trials. For the intrinsic connections, this entailed a decrease in self-inhibition, between 60% (on the left) and 20% (on the right). The remarkable thing about the changes in extrinsic connectivity is that they (both) change from being mildly inhibitory to being excitatory. Quantitatively, these changes were more marked in the right-to-left extrinsic connection. In short, changes in both intrinsic (decreased self-inhibition) and extrinsic (from mildly inhibitory to excitatory) appear to precede stimuli that are subsequently seen.

## 4 Discussion

The aim of this case study was to address two questions: Does prestimulus activity in visually responsive areas in a patient with visual extinction predict subsequent perception (as seen in healthy subjects in other tasks), and do fluctuations in connectivity between these regions determine neuronal and perceptual responses? We used a simple detection paradigm with unilateral and bilateral phase presentation. We concluded that fluctuations in connectivity between regions that exhibited higher activity prior to bilateral seen compared to bilateral unseen trials provide a sufficient account of both baseline fluctuations and perceptual reports. This finding is consistent with studies of normal subjects. However, care should be taken in generalising this conclusion to the normal brain. This reflects the Catch-22 associated with lesion-deficit studies: we can only study the correlates of extinction in the lesioned brain, which means that we cannot exclude the possibility that the physiological (fluctuating connectivity) basis of neuronal and perceptual responses is itself pathological. Having said this, one could argue that the consilience between our results and studies of baseline fluctuations in normal subjects (Fox & Raichle, 2007; Hesselmann et al., 2008a, 2008b) suggests one might find the same changes in connectivity, were it possible to study perceptual extinction in the healthy brain.

### 4.1 Prestimulus activity in visual areas affects stimulus perception

Our results are in line with previous work on visual extinction and the visual areas identified by these. In fact, the two areas that show a higher prestimulus activity prior to bilateral seen trials are very close to the visual areas reported by Rees et al. (2000), when investigating the unconscious residual cortical processing of the extinguished stimulus in the contralesional visual field. We extend the results of previous studies showing that visual areas can be activated without leading to awareness, e.g. (Sarri et al., 2010), by providing evidence for the idea that activity prior to stimulus presentation is indicative for subsequent perception.

Furthermore, the activations in response to unilateral trials in the present study were very similar to the regions reported by Rees et al. (2000) for the same contrast: in both cases, responses in the lesioned right hemisphere were greater and more widespread than in the left (see Fig. 3).

### 4.2 Prestimulus activity in other brain areas might play a role

In addition to the two visual areas, we identified several brain regions that showed signalling differences that were associated with subsequent conscious perception during bilateral stimulation; however, these failed to survive correction for a whole brain search, these failed to survive correction for a whole brain search, i.e. they did not survive FWE correction (possibly reflecting the relatively low efficiency of our single case study). Among these areas are frontal and parietal regions, which have been identified in previous studies of visual extinction (see below). In fact, during stimulus processing the interplay between posterior visual areas such as the ones found here and a fronto-parietal network seems to be crucial for perceptual awareness, e.g. (Driver et al., 2001; Rees et al., 2002a, 2002b; Vuilleumier et al., 2010). In addition, we detected higher prestimulus activity prior to seen bilateral trials bilaterally in the brainstem. This evolutionary old part of the brain is known to control autonomic functions of the peripheral nervous system and modulate arousal and alertness, two criteria that may be important in determining awareness. Indeed, alertness levels are known to modulate the severity of spatial neglect (George, Mercer, Walker, & Manly, 2008; Malhotra, Parton, Greenwood, & Husain, 2006; Robertson, Mattingley, Rorden, & Driver, 1998) and low alertness has even been linked with neglect-like rightward biases in healthy participants (Manly, Dobler, Dodds, & George, 2005) including in extinction tasks (Matthias et al., 2009).

### 4.3 Mechanisms behind visual extinction

We used Bayesian model comparison to investigate potential changes in the coupling between the two visually responsive areas identified prior to the stimulus. We found the highest probability for models that allowed an increase in both intrinsic and both extrinsic connectivity for sensitivity preceding bilateral stimuli that are subsequently seen. In case of the intrinsic connections these changes represented a decrease in self-inhibition. Remarkably, the extrinsic connections changed from being mildly inhibitory to being excitatory. It should be noted, that real extrinsic connections between the two areas are both excitatory (using the neurotransmitter glutamate). However, in DCM, effective connections are polysynaptic and an extrinsic connection can be effectively inhibitory (presumably by targeting inhibitory interneurons). Quantitatively, the changes in effective connectivity were more marked in the right-to-left extrinsic connection, i.e. from the lesioned to the healthy hemisphere. Crucially, these changes in connectivity for sensitivity were sufficient to explain both the differences in baseline activity prior to stimulus onset and the perception dependent differences in stimulus bound responses.

These results suggest that fluctuations in cortical gain or excitability (both to intrinsic and extrinsic presynaptic inputs) may underlie the decreased neuronal response and a failure to perceive stimuli that are subject to extinction. This is interesting in that exactly the same mechanisms – at the synaptic level – are thought to underlie attentional modulation, which may be dysfunctional in extinction. Furthermore, they speak to the precision-dependent explanation for detecting signals based upon predictive coding; in the sense that precision is thought to be encoded by postsynaptic gain (Feldman & Friston, 2010) and that optimising postsynaptic gain corresponds to attention. This is important because the many mechanisms modulating postsynaptic gain include the classical modulatory neurotransmitter systems, originating in the brainstem (see above). A heuristic (and overly simplistic) explanation for these results could be as follows: spontaneous fluctuations in ascending aminergic and cholinergic neurotransmitter systems result in spontaneous fluctuations in the effective gain of neuronal populations in visual cortex, both to intrinsic and extrinsic afferents. If the resulting increases precede a stimulus, then the neuronal responses evoked by stimulus are amplified and gain access to higher hierarchical levels, enabling deeper processing and perceptual inference – and subsequent perception.

### 4.4 Limitations of the study

In this work, our primary focus was on early visual mechanisms that might underlie fluctuations in the perceivability of stimuli. From this perspective, the current case study represents a lesion-deficit model that enables the comparison of seen and unseen stimuli and their physiological correlates. Generalising our conclusions – about the underlying role of intrinsic and extrinsic connectivity – to the normal brain clearly rests on the assumption that both the perceptual and physiological processing of seen and unseen stimuli are quantitatively the same in our patient and the normal population.

One might also argue that our findings would be more plausibly generalised if we had been able to reproduce the results using further patients with extinction. This is certainly the case and extinction has a reasonably high prevalence. However, despite testing several patients with extinction, only the patient reported here was considered suitable for fMRI. Although this is a single case study, one can be reassured by the fact that fMRI produces an enormous amount of data and the degrees of freedom we have used for our analyses were much greater than any conventional group study. Having said this, this case study should probably be regarded as proof of principle, until reproduced in other people.

### 4.5 Methodological aspects

From a methodological perspective, we present a practical example of the use of DCM in a patient with a parietal lesion. Frequently used methods to investigate changes in connectivity are often based on correlations and address changes in so-called functional connectivity, which describes statistical dependencies between spatially segregated neuronal events. However, this approach does not support any conclusions about directionality or the distinction between intrinsic and extrinsic influences. In contrast, effective connectivity is based on a mechanistic model of how the observed data were caused and allows the modelling of directed and reciprocal connections within and between brain areas.

Although DCM is an established procedure; for those people less familiar with the analysis of fMRI time-series, DCM can be contrasted with alternative procedures: in general terms, distributed interactions, as measured by fMRI, can be characterised in terms of either functional or effective connectivity. Functional connectivity refers to the statistical dependence or correlations between observed responses (Biswal, Yetkin, Haughton, & Hyde, 1995; Cordes et al., 2000), while effective connectivity refers to the underlying and directed connections strengths that cause correlations (Friston, 1994). Analyses of effective connectivity generally use dynamic causal modelling, although other techniques have been tried (such as structural equation modelling (SEM), multivariate/vector autoregressive models (MAR/VAR) and Granger causality). Dynamic causal modelling is unique in that it incorporates an explicit model of neuronal interactions and allows for region specific neurovascular coupling. If these regional differences are ignored, they can lead to false inferences about effective connectivity (David et al., 2008). DCM is therefore the only approach that allows one to test hypotheses about connectivity at the neuronal level. More precisely, it uses a neurobiologically plausible model of neural population dynamics and a biophysically plausible forward model which describes the transformation from neural activity to the measured hemodynamic signal (Goebel, Roebroeck, Kim, & Formisano, 2003; Stephan & Friston, 2011). Consequently, it is possible to fit the parameters of the neural and the forward model in a way that predicted time series are optimally similar to the observed ones.

### 4.6 Conclusion

In conclusion, we studied a patient with visual extinction after a right parietal lesion that spared visual cortex. We were able to extend previous work showing that activations in visual, parietal and frontal areas can be observed without awareness, e.g. (Sarri et al., 2010). In doing so, we have tried to infer the mechanisms that determine whether extinction will occur during bilateral stimulation. We found that the prestimulus activity in two visual areas in both hemispheres showed increased activity prior to bilateral seen stimuli compared to those that were unseen. In addition, we used dynamic causal modelling to examine directed changes in coupling within and between these two areas and found that all four intrinsic and extrinsic connections were increased for several seconds prior to stimulus onset. In line with previous studies of prestimulus activity and its role in perception, our results support the idea that prestimulus activity in distinct brain areas is an important determinant of subsequent perception and behaviour.

## Figures and Tables

**Table 1:** Stimulus localiser activated visual areas. Activations during the stimulus localiser for the main contrast left>right are restricted to the right hemisphere, and vice versa. Directions refer to visual fields.

**Left**>**right**	MNI coordinates			t-value	P-value	Cluster size
	x	y	y			
R BA 17 (including calcarine sulcus)	12	−82	0	6.17	<0.0001^a^	107
R BA 19/ occipital medial	34	−84	14	5.03	=0.011^a^	19
R Precuneus	10	−74	60	3.94	<0.0001^b^	159
R Inferior orbital frontal	54	44	−12	3.29	=0.001^b^	4
R Superior occipital	28	−82	46	3.27	=0.001^b^	4

**Right**>**left**						
L BA 17 (including calcarine sulcus)	−8	−80	−2	4.16	<0.0001^b^	84

a Voxel-level statistics, *p*<0.05, FWE.

b Voxel-level statistics, *p*<0.001, uncorrected and a clustersize of at least 10 voxels. *L*=left hemisphere, *R*=right hemisphere, left=left visual field, right=right visual field.

**Table 2:** Activity differences during the extinction paradigm for the baseline period testing for areas that show higher activity before BS compared to BU trials. Voxel-level statistics at *p*<0.001 uncorrected.

	MNI coordinates			t-value	P-value	Cluster size
	x	y	y			
Brainstem R	16	−30	−20	3.54	<0.0001	126
SMA R	4	−22	58	3.39	<0.0001	27
Paracentral lobule R	12	−40	52	3.33	<0.0001	27
Parahippocampal region R	14	−6	−22	3.21	=0.001	11
SMA L	−4	8	54	3.18	=0.001	2
Brainstem L	−10	−28	−22	3.16	=0.001	5
Rectus L	−10	46	−20	3.11	=0.001	1
Frontal medial L	−44	56	18	3.11	=0.001	1
